# Telemedicine during COVID-19 Crisis and in Post-Pandemic/Post-Vaccine World—Historical Overview, Current Utilization, and Innovative Practices to Increase Utilization

**DOI:** 10.3390/healthcare10061041

**Published:** 2022-06-03

**Authors:** Jitendra Singh, April Albertson, Brandi Sillerud

**Affiliations:** School of Nursing & Healthcare Leadership, Minnesota State University Moorhead, Moorhead, MN 56563, USA; aprildaisy1979@yahoo.com (A.A.); brandi.sillerud@mnstate.edu (B.S.)

**Keywords:** telemedicine, COVID-19, workforce shortages, utilization, post pan-demic, telehealth, essential industries

## Abstract

Telemedicine’s underutilization ended when the COVID-19 pandemic caused people to isolate and kept them from seeking healthcare services at their local hospitals and clinics. With the aid of the CARES Act of March 2020, healthcare providers quickly implemented telemedicine services to meet the various needs of their patients. During the pandemic, healthcare systems saw a significant increase in telemedicine visits. Essential industries turned to healthcare providers for assistance in keeping their workers healthy and to maintain production in the country’s critical infrastructure. Telemedicine services could quickly address health concerns, help address industry needs, and combat workforce shortages. As quickly as telemedicine services grew, telemedicine service utilization waned as people started to move closer to a pre-pandemic lifestyle. This descriptive study builds on an in-depth literature review by utilizing a fishbone diagram and SWOT analysis examining the potential factors related to telemedicine underutilization. To promote telemedicine utilization, application of Rogers’ Diffusion of Innovation theory outlines how to gain support for the benefits of telemedicine and build on opportunities brought out by the COVID-19 pandemic. Implication for practice could include establishing virtual clinics for industries plagued with workforce shortages.

## 1. Introduction

COVID-19 wreaked havoc on the working world. Industries struggled to quickly interpret changing guidelines, infection control regulations, and employee illness recommendations while trying to maintain production goals. Industry turned to healthcare organizations for guidance on how to oversee these new challenges and continue to keep their businesses running [1,2].

Essential industries needed their workers on the lines to keep up production. One of the hardest hit industries was the meat packing plants. Workers in tight quarters and needed at each step of the production process and positions filled by migrant and minority workers needing every penny to support their families put extra stress on job attendance and calling into work sick. This contributed to several large COVID-19 outbreaks within this industry.

One highly publicized outbreak occurred in Sioux Falls, SD, at a Smithfield packing plant. The first COVID-19 case occurred on 24 March 2020, and on April 14th the leaders closed the plant to stop the spread of the COVID-19 virus and to thoroughly clean all production space [3]. The Center for Disease Control and Prevention investigated the outbreak and gave over 100 recommendations to the plant to implement prior to reopening. A congressional investigation in April 2021 showed that 42% of the plant’s workers became infected with COVID-19, which was 1674 people, including four deaths. Smithfield was fined $13,494 in November 2021 for the outbreak [4].

The COVID-19 pandemic has opened the eyes of industrial leaders on how a virus can disrupt business operations. Incidences, such as the Smithfield outbreak, highlight the importance of a strong emphasis on the health of workers, starting at the leadership level and becoming engrained in the organizational culture. Close partnerships with local healthcare organizations emphasize this commitment.

Healthcare organizations needed to change their care delivery methods during the pandemic. Telemedicine services quickly became a more utilized method to reach patients and address their needs. This technology provides innovative opportunities for healthcare professionals [5]. This was highlighted in the recently published work where researchers found that telemedicine became an option of choice for providers as the pandemic continued to progress and active cases increased, especially during the second and third waves [6,7].

This leads to an important question: how can healthcare organizations partner with industries to keep their commitment to their workers? One method is to establish locally-based virtual clinics within the industry’s walls. Virtual clinics allow for timely access to healthcare providers without having to leave work. This lessens the time the worker is away from the production line and keeps operations running. Virtual clinics would connect the local healthcare organization and local industries, allowing them to partner closely while navigating through the industry’s healthcare needs.

While telemedicine usage increased at the start, through the first wave of COVID, latest trends suggest a decline in utilization of services. Beginning with an overview of telemedicine technology, this study examines factors behind the declining use of telemedicine and examines how this technology could be used to reduce the shortage of healthcare workforce. Secondary aims include completion and analysis of strengths-weaknesses-opportunities-threats (SWOT), the fishbone diagram, and an explanation of the entire phenomenon (usage and decline of telemedicine) using Roger’s Diffusion of Innovation theoretical framework.

## 2. Literature Review

Since its inception in the 1950s, telemedicine has shown great promise as a healthcare delivery method. The first uses connected specialized care located in urban areas to rural areas. Neurologic and radiology exams could be sent to specialists in other areas for interpretation, while psychiatric consultations could be performed at distance locations [8].

This technology not only connects specialty care to rural areas, but also brings healthcare to those in need, saves time and resources, supports rural providers, and can be provided in various settings, including workplaces. The research showed that patients spending less time traveling to a healthcare center will have an increased ease in receiving care and keeps their healthcare a top priority in their lives [9].

*Global Telemedicine Utilization* With the birth of the internet in the 1990s, the reach of telemedicine services has grown exponentially. A study conducted in Senegal showed how the use of telemedicine technology in remote areas can provide support and guidance to isolated practitioners in rural areas. This additional support increased recruitment of practitioners to these underserved regions [10].

A western African study looked at the reasons behind the resistance to telemedicine utilization during the Ebola crisis. Results showed resistance occurring when practitioners felt the technology could harm their autonomy. Further research showed that government support creates an increased trust of telemedicine technology [11]. Without this government backing, both practitioners and patients will be less likely to use telemedicine services.

*Telemedicine Utilization during the COVID-19 Pandemic* Telemedicine visits increased 10-fold in comparison to pre-pandemic levels for some healthcare systems during the COVID-19 pandemic [12]. The CARES Act of March 2020 implemented policy changes allowing for quick implementation of telemedicine services. These changes included payment parity, relaxed licensing regulations, decreased auditing, and allowing popular virtual connection applications for visits [13].

The quick implementation helped bridge the gaps in care caused by the COVID-19 pandemic. Providers could address the most acute and chronic conditions via telemedicine technology. A study of 3454 US households showed 50% of them using telemedicine services during the pandemic, with 86% reporting a positive experience [14].

With its increased utilization, telemedicine applications broadened to meet the care needs of patients in various locations, including in the workplace. This allowed for no travel to a provider’s office and more convenient scheduling and allowed workers to be more efficient with their time. A Hungarian study showed that an organization’s healthcare support for their workers led to increased employee satisfaction and loyalty [15].

While telemedicine has several advantages, one must also note that its utilization quickly declined as people returned to a pre-pandemic lifestyle. Building on historic and recently published evidence, this study aims to conduct a thorough investigation into expansion of telemedicine during COVID, followed by a rapid decline in services once some level of “normalcy” was achieved. Quality Improvement tools, such as SWOT analysis and the fish bone diagram, have been used to further illustrate the problem or declining use of telemedicine. To reverse the decline in utilization and sustain use going forward, telemedicine will need government support through CMS data gathering and permanent policy changes, industry commitment to employees’ health, and a renewed adoption process. Efforts have been made to focus on these key issues while conducting SWOT analysis and fish bone analysis.

Research on telemedicine has focused on its benefits and increased utilization; however, there is a scarcity of literature that examines the reasons behind the declining use of telemedicine, especially through a Quality Improvement (QI) lens. Additionally, there is a lack of work in which a theoretical knowledge base has been utilized to explain the early rise in telemedicine services during the pandemic and the decline in utilization post-pandemic. Because there is still a scarcity of research on the topics highlighted above, this project could both contribute to the growing evidence in the field of telemedicine and have wide practical applications. Dissemination of findings could help healthcare leaders, administrators, and clinicians as they advance towards implementing telemedicine or similar services to serve patients and families.

## 3. Objectives

### Objectives of the Study

- Review history, evolution, and utilization of telemedicine technology.
- Conduct SWOT analysis of telemedicine technology utilization.
- Review telemedicine technology utilization during the COVID-19 pandemic.
- Using Rogers’ Diffusion of Innovation Theory and the fishbone diagram, highlight reasons for decline in telemedicine utilization.
- Application of Rogers’ Diffusion of Innovation theory to increase telemedicine utilization.
- Provide evidence-based recommendation to combat workforce shortages by implementing telemedicine strategies.

## 4. Materials and Methods

This descriptive study highlights the use of telemedicine technology during the COVID-19 pandemic, reasons behind its recent decline in utilization, and the opportunities that telemedicine technology utilization provides for combatting workforce shortages. The history of telemedicine technology is reviewed, utilizing a timeline. The evolution of technology is brought forth through research. A SWOT analysis pinpoints opportunities that this care delivery method offers and barriers it has faced in doing so. This study shows how the COVID-19 pandemic response quickly broke down those barriers, allowing telemedicine to become a part of daily care delivery, and how it impacted problems that the pandemic presented to healthcare and business organizations. A fishbone diagram provides reasons for the decline in telemedicine utilization in the later part of the COVID-19 pandemic. Rogers’ Diffusion of Innovation Theory is analyzed and applied regarding underutilization, and suggestions for application to increase utilization are presented. Finally, an evidence-based approach is presented to utilize telemedicine services to combat workforce shortages. A thorough study was developed by using scholarly search engines, peer-reviewed journal articles, government reports, and other academic resources publicly available to researchers and scholars.

## 5. History and Evolution of Telemedicine

**Figure 1:**
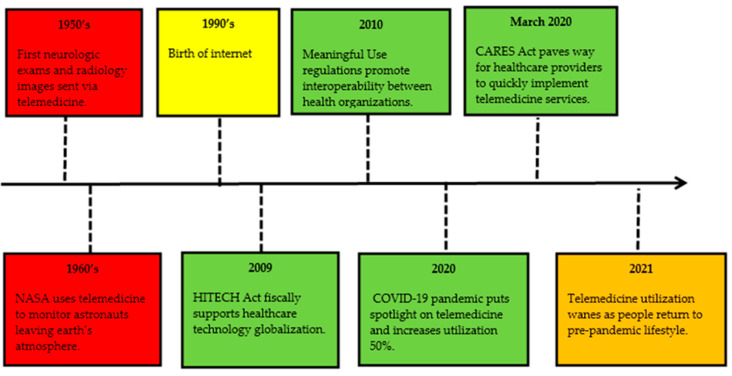
Timeline of telemedicine evolution.

Figure 1 shows that modern telemedicine use can be traced back to the late 1950s, with the University of Nebraska sending neurologic exams via interactive telemedicine and Canadian radiologists sending images by coaxial cable. In 1960, the Nebraska Psychiatric Institute and Norfolk State Hospital connected for consultations. Other organizations began utilizing this technology to send X-rays, stethoscope sound, fluoroscopy images, and electrocardiograms. These early uses helped connect rural areas to more specialized care located in urban areas [8].

The STARPAHC (Space Technology Applied to Rural Papago Advanced Health Care) project in the 1960s allowed technology intended for use for NASA astronauts to be used to connect a Native American reservation to medical care [8]. This project was instrumental in connecting the patient to the healthcare provider and opening doors for many populations to receive care at any location. Other examples of utilization include connecting to soldiers in war zones, remote research stations in the Antarctic, and inmates at prisons [8].

The 1990s brought the biggest impact on telemedicine services, with the birth of the internet. The internet transformed how healthcare providers could connect with patients, colleagues, and experts around the globe. Internet infrastructure allowed for increased communication speeds and storage, security, and digitizing of information, which aided healthcare organizations in building software applications to improve their operations and increase their quality-of-care delivery [8].

The 2009 HITECH (Health Information for Economic Health) Act earmarked money to achieve globalization and assist healthcare organizations to deploy the technology needed to reach these connection goals. The Centers for Medicare and Medicaid Services (CMS) developed Meaningful Use regulations in 2010 to promote interoperability between healthcare organizations, while the Affordable Care Act created Accountable Care Organizations that pushed the maturation of telemedicine services [16]. The government support of growing technology utilization in healthcare showed both providers and patients that the future of healthcare depends on a strong technology infrastructure.

The COVID-19 pandemic brought telemedicine into the spotlight and transformed it into a daily care delivery method for various areas of healthcare. In February 2020, the Center for Disease Control (CDC) offered guidance advising persons and healthcare providers to practice social distancing and utilize telemedicine technology to provide healthcare services [17]. This guidance created hesitation in those needing healthcare for non-acute needs.

The March 2020 Coronavirus Aid, Relief, and Economic Security Act or CARES Act bill earmarked $2 trillion in relief for industries, local governments, public health, and individuals impacted by COVID-19 pandemic. The Act broke down barriers, allowing healthcare providers to quickly implement telemedicine services. The US Department of Health and Human Services (HHS) gave notice to providers that they may use popular applications that allow video chats, for example Google Duo, FaceTime, and Zoom. The Act also relaxed licensing regulations and addressed reimbursement parity [18].

Telemedicine encounters between January and March 2020 increased by 50% in comparison to the same period in 2019 [8]. Services expanded beyond specialist visits to primary care visits, therapy visits, nursing home rounds, hospital inpatient rounds, and more. As quickly as tele-medicine services grew during the pandemic, 2021 showed a drastic de-cline in utilization as people went back to pre-pandemic lifestyles.

## 6. SWOT Analysis of Telemedicine Services

**Figure 2:**
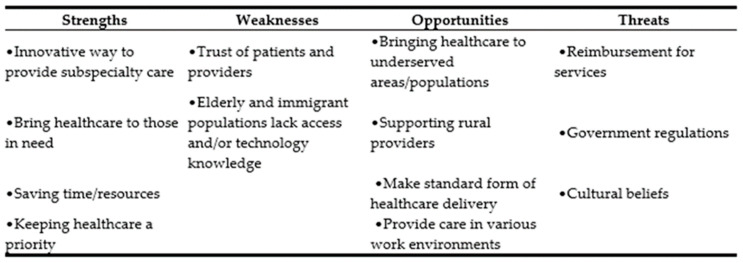
Strengths, Weaknesses, Opportunities, Threats (SWOT) Analysis.

Telemedicine services were an underutilized option for healthcare delivery up until the COVID-19 pandemic. What factors contributed to this underutilization? Figure 2 SWOT analysis outlines the reasons behind this and the strengths and opportunities for current and future use.

### 6.1. Strengths

A UK 2011–2012 study conducted 24 interviews with 29 participants in both “live” and “mock-up” telemedicine stroke assessments. “I think it’s brilliant…it feels like they’re in the room”, stated one participant. To reduce permanent effects or death, stroke patients must receive specialized care in a short timeframe from the onset of stroke symptoms. The use of telemedicine allows those experiencing strokes in more remote and medically under-served areas to benefit from stroke-specialist diagnosis and care [19] (p. 108).

Telemedicine services are not limited to specialist care; family medicine, behavioral health, and occupational medicine are other areas using technology. Connecting medically underserved rural areas is beneficial, but urban areas can benefit as well. Easier access to care fits busy schedules, connecting at the workplace, home life, or the daily commute and keeps an individual’s health a priority.

Using these services saves time and money for both the provider and patient. Less resources are utilized to provide telemedicine services versus a traditional office visit, for example, there is no need to clean the exam rooms. Patients spend less time traveling to a healthcare center and have an increased ease in receiving care [9]. These benefits allow patients and providers to keep healthcare a high priority in the lives of those utilizing this care delivery method.

### 6.2. Weaknesses

Technology brings many benefits, but technology can also bring resistance. A 2013 Iranian study showed 96.1% of clinicians had little knowledge about telemedicine. This limited knowledge influenced their perception of technology. Proper understanding of telemedicine technology, particularly by physicians, will lead to successful implementation and deployment of the technology [20].

Patient perception is important as well. A 2017 UK focus group of people ages 65–76 showed eagerness and willingness to learn innovative technology, but also showed barriers. The barriers were categorized into several areas, including cost, decreased knowledge and confidence, lack of instruction, and health-related factors [21].

The perceptions of telemedicine technology of both providers and patients are positive, but both parties experience resistance due to a lack of knowledge of how to utilize the technology. Lack of knowledge drives down willingness of utilization [20]. Anyone experiencing barriers to technology, whether it is related to lack of instruction or cost, will be less likely to seek healthcare through this delivery method.

### 6.3. Opportunities

Since the inception of the internet in the 1990s, the reach of telemedicine services has grown exponentially [8]. Providing healthcare services in rural/remote areas around the world is now a possibility. Telemedicine services not only provide needed care to isolated areas, but also provide support to healthcare providers that practice in these areas.

A study conducted in Senegal showed an uneven distribution of physicians in the country. A total of 71% practiced in the capitol city of Dakar, leaving remote areas underserved. The country undertook several measures to increase the recruitment of physicians to these underserved regions. One measure was to implement telemedicine services to allow the rural practicing physicians to get advice from experts, reduce their professional isolation, and decrease the feelings of work overload. A survey of 60 physicians resulted in 60% feeling that this telemedicine initiative would positively impact recruitment to these isolated areas [10].

The broad reach of telemedicine services allows an increase in the scope of services provided. The ability to connect whenever and wherever increases the opportunities for utilization. Stepping off the manufacturing line for a worker’s compensation visit for a sore back, a therapist appointment over a lunch hour, and a quick urgent care visit for a child with a sore throat are examples of how telemedicine can become a standard of care.

### 6.4. Threats

If healthcare providers can reach patients anywhere and at any time, why have telemedicine services not become a standard of care? Despite its recent growth, telemedicine deals with several barriers, challenging more extensive adoption. Regulation concerns include service reimbursement, information privacy and security, provider licensing, and liability [22]. Another concern, due to the impeded growth of telemedicine, is the lack of quality data for this method of healthcare delivery.

The success of telemedicine often relies on the behavioral and cultural beliefs of the healthcare provider and patients. A western African study looked at reasons behind the resistance to the use of telemedicine during the Ebola crisis. Utilizing the Technology Threat Avoidance Theory (TTAT), the researchers found that the resistance to telemedicine can be explained by the healthcare providers’ perceived threat of and ability to control this care delivery method. Resistance occurs when providers feel this technology could harm their autonomy [11].

In African countries lacking social means, government support is instrumental. A study showed how this support was found to not only reduce anxiety and costs but reduce resistance by weakening the effects of the perceived threat and lack of control [11]. Thus, government support creates increased trust of this technology.

## 7. Problems Posed by COVID-19

### 7.1. Healthcare Delivery

According to the CDC, the COVID-19 pandemic caused a 40% increase in delayed or avoided medical care for American adults [23]. The risk of acquiring COVID-19 from in-person visits to clinics or hospitals outweighed the benefits of seeking care. This results in missed screenings, missed follow-ups for chronic diseases, delays in elective procedures, and delays in care for more critical illnesses. With patients avoiding hospitals and clinics, how can they keep their health a priority?

Healthcare delivery shifted from preventative medicine to urgent care visits, emergency room visits, and hospitalizations. Healthcare providers were overwhelmed with COVID-19 response, daily recommendation changes, and trying to stay healthy themselves. In April 2020, CMS recommended all elective procedures be halted to conserve personal protective equipment (PPE), hospital beds, ventilators, and hospital staff.

These shifts in healthcare priorities create another challenge. How will healthcare get back to “normal”? In the upcoming transition period following the pandemic, the number of elective procedures and delayed appointments will put additional pressure on healthcare systems and outstrip availability [24].

### 7.2. Workforce Shortages

The COVID-19 pandemic caused a domino effect on workforce shortages: Starting with someone getting COVID-19, someone exposed to that person, a child in daycare getting COVID-19, daycare children exposed to that child, older child getting COVID-19, school children exposed to that child, daycares closing, schools closing, workplaces sending workers home for symptoms and/or exposure; the list goes on and on. Just one person getting COVID-19 can have a ripple effect that can shut down a business and lead to a workforce shortage.

Every sector of global industry was affected in one way or another by this pandemic. Governments began labeling businesses as “essential” and “non-essential”. Those deemed “essential” were expected to continue operations to keep necessary services going. One industry high on the “essential” list were the meat packing plants. The meat industry is vulnerable to viruses spreading due to the physical work environment and labor intensity. High humidity and lower temperatures during processing increase virus survival and proliferation [25].

Besides the environmental factors contributing to the spread of COVID-19 in these plants, sociodemographic and workforce factors contributed as well. Young, insecure, and poorly paid workers felt discouraged from reporting any symptoms due to fear of penalty, which played a part in spreading the virus. These companies often relied on migrant workers who not only worked in tight quarters, but also lived in overcrowded housing and relied on overcrowded transportation to and from work [26]. At the Smithfield plant in Sioux Falls, SD, 40 different languages are spoken. When the COVID-19 outbreak hit, those processing plant workers showing symptoms were distributed English written informational packets [27].

In October 2021, Debbie Burkowitz, a safety and health expert, testified in front of a Congressional House Subcommittee on the impact COVID-19 had on meat packing plants. “More workers have died from COVID-19 in the last 18 months in the meat and poultry industry than died from all work-related causes in the industry in the past 15 years”, said Burkowitz. In the first year of the pandemic, 59,000 meat processing plant workers were infected with COVID-19, with 269 workers dying from the virus [28].

## 8. Ways Telemedicine Utilization Resolved COVID-19 Problems

### 8.1. Healthcare Delivery

COVID-19 was the spark that telemedicine care delivery needed to be brought into daily utilization. Telemedicine visits increased 10-fold in comparison to pre-pandemic levels for some healthcare systems [12]. How did this care delivery method that has been around for decades, along with a lengthy list of implementation barriers, become a standard of care so quickly?

The Public Health Emergency Declaration from the Department of Health and Human Services, along with the CARES Act of March 2020, broke down barriers to allow for healthcare leaders to implement telemedicine services [13]. Policy changes allowed providers to quickly address the mounting gaps in care. These changes included:

- Medicare paying for telemedicine services furnished to beneficiaries at the same rate as in-person visits.
- Department of Health and Human Services not auditing whether there was a prior patient-physician relationship established.
- Many state governors relaxing licensure requirements, allowing providers to see patients located anywhere within the United States.
- HIPAA (Health Insurance Portability and Accountability) regulations relaxing, allowing several popular virtual connection applications to be utilized for telemedicine connections.

Healthcare organizations were able to quickly convert in-person visits for medication follow ups, urgent care visits, mental health therapy, physical therapy, consultations, and more to a virtual platform. This allowed providers and patients to negate the fear of coming into a healthcare facility and being potentially exposed to COVID-19. Sharp Rees Stealy Medical Centers in San Diego, CA, USA, performed 70% of all visits via telemedicine technology during the peak of the pandemic [29].

The quick implementation of telemedicine services helped to bridge the gap in care. Providers were able to address the most acute and chronic conditions without having the patient step foot in the building. A study of 3454 US (United States) households showed that 50% of the households used telemedicine services due to the inability to obtain in-person care. Those utilizing telemedicine reported an 86% positive experience [14].

### 8.2. Workforce Shortages

Telemedicine visits allowed for a better use of healthcare resources, from less staff involved and/or staffing time, or resources for cleaning exam rooms, and increasing the ability to work remotely. Telemedicine can be a more efficient method of care delivery for healthcare organizations. These efficiencies permitted stretched resources to better meet the needs of patients.

Telemedicine technology connects providers to patients anywhere. Visits can also be conducted at various times of the day without the need for travel time. Therapy appointments over a lunch break, a quick acute visit before sending a child to school, or a worker’s compensation assessment from the workplace are all examples of how telemedicine can reach patients where and when the care is needed and keep people working and more efficient in their day.

Research conducted in Hungary focused on data analysis of two-layers of factors that impact employee loyalty, well-being, and satisfaction. Mental and emotional health or ‘internal locus of control’ variables contribute to workplace wellbeing with no impact on employee loyalty or satisfaction. The ‘external locus of control’ variables, such as healthcare support, led to employee satisfaction and loyalty [15].

Using telemedicine technology can support workplace health promotion (WHP) projects. The WHP life cycle model developed by researchers is based on the World Health Organization (WHO) model of a healthy workplace constant improvement process. These WHP projects are integral in organizations wanting to improve workplace health. The life cycle model includes process and procedure analysis in the organization, developing interventions to support individuals desiring a change in their personal health and creating organizational interventions to produce better working conditions [30].

Workplace commitment to improving the health of their workers can increase employee satisfaction and reduce turnover. Industries combating workforce shortages should focus efforts in this direction. Incorporating employee health into the company’s culture will benefit all.

## 9. Post-COVID-19 Decline in Telemedicine Utilization

Telemedicine utilization quickly declined as people returned to pre-pandemic life. Healthcare visits returned to clinics and hospitals, with stretched, fatigued healthcare workers trying to catch-up on missed screenings and delayed appointments. Instead of continuation of telemedicine technology to save on healthcare resources, the technology was pushed again to the back burner. Why did the overtaxed healthcare industry turn away from the resource-saving delivery method?

### 9.1. Fishbone Analysis

A fishbone diagram is a visual aid helping identify the causes or reasons contributing to a problem. The causes are categorized and then broken down into each specific reason attributing to the overall problem. The overall problem is included in the box on the far right of the diagram, and each category is located on a diagonal line or fishbone [31].

**Figure 3:**
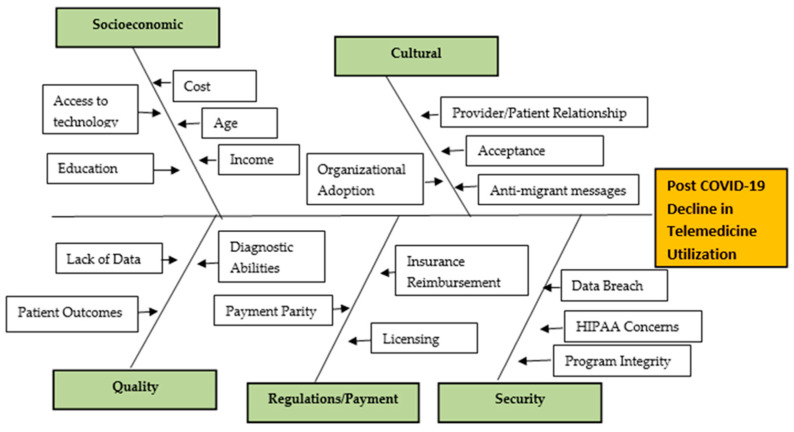
Fishbone analysis.

The fishbone diagram in Figure 3 is used to show the reasons attributing to the decline in telemedicine utilization as the COVID-19 pandemic declines and services begin to normalize.

#### 9.1.1. Socioeconomic Related Issues

Socioeconomic issues that may contribute to telemedicine utilization decline include lack of access to technology, cost of technology, lower education level, and lower income. A study conducted in 2020 utilizing data from the 2017 China Migrants Dynamic Survey (CMDS), a multivariant regression analysis, looked at the connection between health-seeking behavior and socioeconomic status (SES). This study showed a significant connection between education and income and healthcare-seeking behavior, while occupation had no significant impact [32].

#### 9.1.2. Cultural Related Issues

Cultural related issues can create barriers for those seeking telemedicine services. Anti-migrant messaging focuses on migrants being a burden to the health system rather than structuring inclusive policies to help integrate migrants as members of our communities and allocating sufficient funding to provide intersectoral collaboration [33]. Another factor is the impact that the patient/provider relationship may sustain through telemedicine services rather than in-person visits and the potential negative affect it may have on establishing a trusting relationship [34]. Organizations must support telemedicine services and provide education for both providers and patients. Understanding how to use the technology provides a solid foundation for utilization [10].

#### 9.1.3. Quality Related Issues

The rapid adoption and utilization of telemedicine services allowed providers to continue to meet the needs of their patients. The Centers for Medicare and Medicaid (CMS) are now assessing the quality of care provided through telemedicine services to make future decisions through regulatory action [35]. Challenges also exist in performing traditional patient examinations through telemedicine, which could potentially lead to poor patient outcomes or inability to provide an accurate diagnosis [36].

#### 9.1.4. Regulations/Payment Issues

Regulatory barriers, such as payment parity, insurance reimbursement, and provider state licensing laws, hinder telemedicine success [36]. The March 2020 CARES Act temporarily broke down these barriers, allowing providers to obtain equal payment for telemedicine services versus seeing the patient in-person. The Act also allowed providers to see patients in locations outside the providers licensed state. Currently, permanent regulatory changes are undecided.

#### 9.1.5. Security Related Issues

Telemedicine services raise the concern of the security of personal health information. With these services being conducted online, they are vulnerable to cybersecurity attacks. Additionally, lack of privacy policies and lack of understanding of these policies may turn patients away from the service [36]. Telemedicine services can be performed anywhere, which raises concern for Health Insurance Portability and Accountability Act (HIPAA) breeches.

## 10. Discussion

### 10.1. Theoretical Framework Rogers’

Diffusion of Innovation Theory can help explain how an idea or product gains popularity and support and then diffuses (spreads) through a population group. The population adopting the idea or product is the goal of the Diffusion of Innovation Theory. The person must perceive the idea or product as innovative or new for adoption to occur [37].

Adoption does not happen all at once. Characteristics differ from people who adopt early to people who adopt late. There are five categories of adopters (Innovators, Early Adopters, Early Majority, Late Majority, and Laggards), with most of the general population falling into the middle categories (Early and Late Majority). When promoting innovation to a specific target population, the characteristics of that group must be considered when developing strategies to gain adoption [37].

### 10.2. Adoption Process Related to SWOT

Analysis Innovators and Early Adopters both embrace new opportunities. They understand the need for change and look for the Strengths and Opportunities of the idea or product. These groups are often leaders in the organizations and their early adoption is key to moving the idea forward to others. The excitement of quickly solving a problem, such as how to bring healthcare to those isolated at home, appeals to the early adopters, who then start the implementation process.

Those in the later adopters’ groups (Early Majority, Late Majority, and Laggards) focus on the Weaknesses and Threats of an innovation. They need to see evidence of the innovation at work. Early adopters will need to provide data on how many people have tried the innovation, statistics, and outcomes are needed to help gain adoption from these groups. Patient testimonials on the ease of use and provider testimonials on the ability to diagnose and meet patient care needs would aid in the adoption of telemedicine services.

### 10.3. Adoption Process Related to Fishbone Analysis

The Fishbone Analysis focuses on how telemedicine innovation hit roadblocks or barriers. Instead of moving forward with more adoption and increased utilization, tele-medicine service utilization declined. The analysis laid out five categories of contributing factors to the decline, including socioeconomic, cultural, quality, regulations/payment, and security.

Each person in the adopter categories may be affected differently by the fishbone analysis categories. Fishbone analysis is also known as a cause-and-effect diagram [31]. For example, a lower income person who cannot afford the technology to seek healthcare through telemedicine would be less likely to be an adopter. These individuals have additional factors that may influence their ability to adopt innovation. Leaders must understand the reasons behind groups failing to adopt an innovation and create systems to address or minimize barriers.

### 10.4. Adoption Process Related to Healthcare Industry

The healthcare industry quickly implemented telemedicine services during the pandemic, not giving time for full adoption of the new care method. As quickly as the barriers came down, the healthcare industry understands that those barriers can come back just as quickly. For the healthcare industry to normalize telemedicine going forward, permanent policy changes need to take place. Healthcare leaders will then need to implement telemedicine services in a new way that brings staff through the adoption process and provides more sustainable telemedicine utilization. Innovators and Early Adopters can lead the way on promoting permanent policy changes that will positively impact the uptake and utilization of telemedicine. The goal should be focused on establishing the parity of care with in-person and virtual visits.

### 10.5. Adoption Process Related to Essential Industry

Essential industries must use the pandemic as a learning tool going forward. Keeping a strong partnership with local healthcare organizations will aid in developing the commitment to a healthy workforce and combat workforce shortages. The adoption process will come easier if industry leaders engage workers and together commit to a healthy work environment. Leaders should collaborate with workers who are in the Early Majority group to help diffuse the innovation of virtual care. This group of workers can help persuade their colleagues to accept and utilize telemedicine as their preferred healthcare delivery method.

## 11. Recommendations

### 11.1. Virtual Clinic Implementation

This descriptive study yields evidence that continued partnerships with essential industries and local healthcare organizations can benefit all involved. One recommendation for continued partnership is setting up virtual clinics in essential industries to combat workforce shortages. Research shows that a company’s commitment to a healthy work environment can lead to a more committed workforce. The COVID-19 pandemic showed essential companies the importance of their workers’ health. Relying on the resources of the local healthcare organizations allowed companies to maintain essential infrastructure.

Healthcare leaders must build upon the momentum gained during the pandemic and continue the utilization of telemedicine technologies. Telemedicine technology has vast utilization opportunities and will continue to grow as technology advances. For increased adoption of telemedicine services to occur, healthcare leadership will need to focus on the reasons behind the decline in utilization. It is recommended that industry leaders partner with healthcare systems to pilot virtual clinics to gain data and information on patient outcomes and work through the hesitations or issues that the late majority or laggard groups may have about the technology. This data will help those who are reluctant to adopt and provide needed evidence for permanent regulatory and policy changes. Listed below are potential benefits of virtual clinics in essential industries.

#### 11.1.1. Healthcare Industry Implications/Benefits

- Normalize telemedicine as a healthcare delivery method.
- Reduce strain on healthcare resources.
- Build upon innovative technologies available.
- Renew business partnerships.
- Reduce unnecessary Emergency Department visits outside work hours.
- Bridge healthcare gaps with specific populations, e.g., migrant workers.

#### 11.1.2. Essential Industry Implications/Benefits

- Solidify commitment to employee health.
- Normalize healthcare as a priority.
- Increase employee satisfaction and loyalty.
- Reduce employee absenteeism and workforce shortage.
- Maintain production goals.

## 12. Limitations

Because of the descriptive nature of the project, researchers primarily relied on the existing literature and evidence that was published in the past year and historical data/information as it pertains to telemedicine utilization. While the methodology. including the fishbone diagram, SWOT, and Roger’s Diffusion of Innovation, can be utilized in similar situations and at other institutions, these findings may not be generalizable worldwide. Researchers must make efforts to find effective methods to enhance telemedicine usage in the post-vaccine and post-pandemic world.

## 13. Conclusions

The aim of this descriptive study was to examine history, evolution, and telemedicine utilization by conducting SWOT and fish bone analysis with the utilization of a theoretical framework to suggest methods to enhance telemedicine usage. The connection between telemedicine to combat workforce shortage was further explored. Findings clearly indicated that telemedicine services emerged as one of these innovative solutions. The pandemic also created a closer bond between essential industries and healthcare organizations as industries tried to navigate through this crisis. Although telemedicine utilization has waned post-pandemic, it is important to foster these partnerships to maintain healthy workforces going forward. The utilization of Rogers’ Diffusion of Innovation could help leaders to increase utilization of telemedicine post-pandemic. One solution is the partnership of local industries and healthcare systems to pilot virtual clinics. These clinics can help deliver healthcare to essential workers and potentially combat workforce shortages in a post-vaccine/post-pandemic world.

## Data Availability

Not applicable.
